# Extraction Optimization, Physicochemical Characteristics, and Antioxidant Activities of Polysaccharides from Kiwifruit (*Actinidia chinensis* Planch.)

**DOI:** 10.3390/molecules24030461

**Published:** 2019-01-28

**Authors:** Qiao-Hong Han, Wen Liu, Hong-Yi Li, Jing-Liu He, Huan Guo, Shang Lin, Li Zhao, Hong Chen, Yao-Wen Liu, Ding-Tao Wu, Shu-Qing Li, Wen Qin

**Affiliations:** College of Food Science, Sichuan Agricultural University, Ya’an 625014, China; hqhlzy@163.com (Q.-H.H.); wlsicau@163.com (W.L.); hongyi45@163.com (H.-Y.L.); hjingliu@163.com (J.-L.H.); ghscny@163.com (H.G.); slsicau@163.com (S.L.); zhaoli0608@126.com (L.Z.); chenhong945@sicau.edu.cn (H.C.); lyw@my.swjtu.edu.cn (Y.-W.L.)

**Keywords:** *Actinidia chinensis* Planch., polysaccharide, ultrasonic-assisted extraction, microwave-assisted extraction, chemical structure, antioxidant activity

## Abstract

In order to evaluate effects of extraction techniques on the physicochemical characteristics and antioxidant activities of kiwifruit polysaccharides (KPS), and further explore KPS as functional food ingredients, both microwave-assisted extraction (MAE) and ultrasonic-assisted extraction (UAE) were optimized for the extraction of KPS. Furthermore, the physicochemical structures and antioxidant activities of KPS extracted by different techniques were investigated. The optimal extraction conditions of UAE and MAE for the extraction of KPS were obtained by response surface methodology. Different extraction techniques significantly affected the contents of uronic acids, molecular weights, molar ratios of constituent monosaccharides, and the degree of esterification of KPS. Results showed that KPS exhibited remarkable DPPH and ABTS radical scavenging activities, and reducing power. The high antioxidant activities observed in KPS extracted by the MAE method (KPS-M) might be partially attributed to its low molecular weight and high content of unmethylated galacturonic acid. Results suggested that the MAE method could be a good potential technique for the extraction of KPS with high antioxidant activity, and KPS could be further explored as functional food ingredients.

## 1. Introduction

When biologically reactive species such as reactive oxygen species (ROS) prevail over natural antioxidative defenses of the organism, oxidative stress emerges. The biomacromolecules (such as lipid, protein, and DNA) in the human body might be attacked by these ROS, and this can eventually cause many serious complications such as cancer and Alzheimer’s and cardiovascular diseases [1]. Synthetic antioxidants are used in the food industry to inhibit the overproduction of ROS, but they are restricted use due to their potential hazards to health [2]. Recently, the polysaccharides in natural sources have been noticed as novel potential antioxidants due to their low toxicity and high level of antioxidant capacity, such as their radical scavenging abilities [3].

Kiwifruit (*Actinidia chinensis* Planch.), which belongs to the *Actinidia* family, is one of the most valuable fruit crops for its flavor and nutritional qualities. Kiwifruit originates in China and it is widely cultivated in many countries around the world such as China, Italy, New Zealand, Chile, and Greece [4]. It is widely believed that the consumption of kiwifruits has a preventive effect against cardiovascular disease and obesity [5]. Indeed, previous studies have shown that kiwifruit possesses multiple activities and antioxidant activity is one of the most important [6]. Phenolic compounds and vitamin C have been identified as natural antioxidants of kiwifruit [7]. However, the chemical structures and antioxidant activities of polysaccharides, which are abundant in kiwifruit [8], have seldom been investigated. Therefore, the investigation of chemical structures and antioxidant activities of kiwifruit polysaccharides (KPS) is necessary.

Extraction techniques exert significant influences on the extraction yields, chemical structures, and bioactivities of natural polysaccharides [9]. Hot water extraction (HWE) is the traditional method for polysaccharides extraction, because it can easily accelerate the diffusion rate of polysaccharides and improve the extraction efficiency with the increase of water temperature. Nevertheless, HWE has some disadvantages, including high extraction temperature, long extraction time, and a large consumption amount of solvents and energy [10]. Therefore, some physical techniques which could facilitate the extraction process have been taken into consideration. Microwave-assisted extraction (MAE) and ultrasonic-assisted extraction (UAE) have been used to extract polysaccharides from natural sources by reducing energy and time while obtaining higher bioactivities [11]. Indeed, several studies have demonstrated that natural polysaccharides extracted by MAE and UAE exhibit higher antioxidant activity than that of HWE [12,13,14,15]. However, to the best of our knowledge, the optimization of microwave-assisted extraction and ultrasonic-assisted extraction of KPS, as well as the effects of different extraction methods on their physicochemical characteristics and antioxidant activities, have seldom been investigated. Therefore, both MAE and UAE were firstly optimized for the extraction of KPS. Furthermore, the physicochemical characteristics and antioxidant activities of KPS extracted by different techniques (MAE, UAE, and HWE) were systemically investigated and compared.

## 2. Results and Discussions

### 2.1. Extraction Optimization of Polysaccharides from Kiwifruit

#### 2.1.1. Microwave-Assisted Extraction of KPS

**Figure 1:**
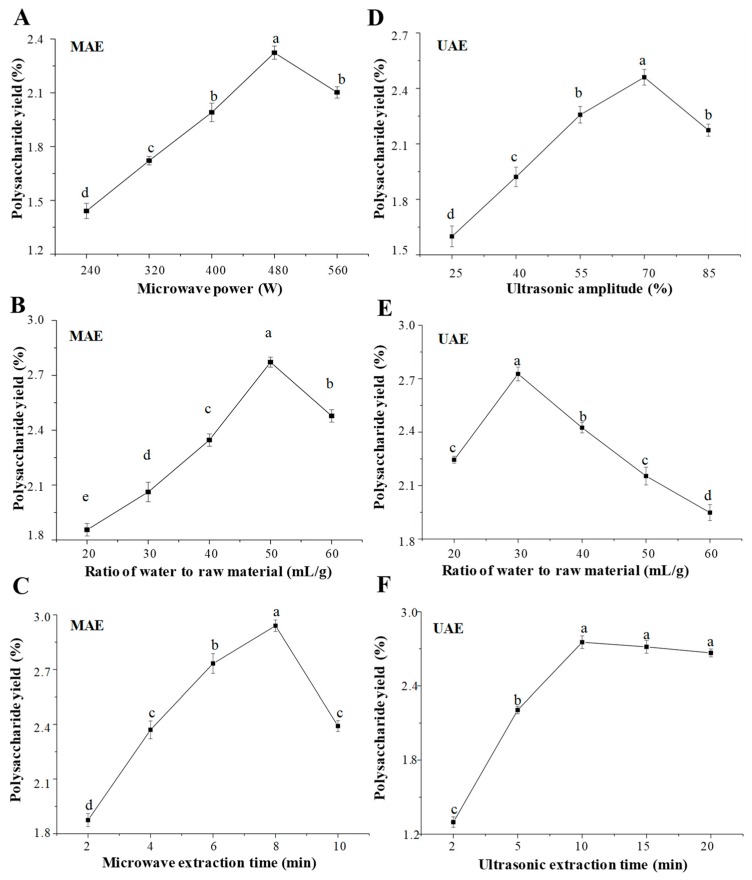
Effects of different extraction parameters of microwave-assisted extraction (MAE) and ultrasonic-assisted extraction (UAE) on the yields of polysaccharides extracted from kiwifruit. (**A**–**C**) the microwave power, ratio of water to raw material, and microwave extraction time of microwave-assisted extraction; (**D**–**F**) the ultrasonic amplitude, ratio of water to raw material, and ultrasonic extraction time of ultrasonic-assisted extraction; The error bars are standard deviations; significant (*p* < 0.05) differences are shown by data bearing different letters (a–e); Statistical significances were carried out by ANOVA and Ducan’s test.

The microwave power, extraction time, and ratio of water to raw material are significant parameters to affect the extraction yield of polysaccharides [16]. The effects of microwave power, ratio of water to raw material, and extraction time on the yield of KPS are shown in Figure 1. Briefly, the yield of KPS increased as the microwave power increased from 240 to 480 W and then decreased when the microwave power was above 480 W (Figure 1A). This could imply that the enhancement of microwave power could accelerate the mass transfer of intracellular substances. However, too high microwave power could lead to the degradation of polysaccharides [10]. The extraction yield of KPS increased rapidly as the ratio of water to raw material increased from 20 to 50 mL/g and reached the highest extraction yield at 50 mL/g (Figure 1B). However, the extraction yield of KPS decreased significantly while the ratio of water to raw material continued to increase. Furthermore, the extraction yield of KPS increased with the increase of microwave extraction time from 2 to 8 min, and reached a maximum yield at 8 min (Figure 1C). When the extraction time increased further, the extraction yield decreased sharply. This could imply that excessive extraction time with microwave irradiation would lead to the degradation of polysaccharides [10]. Finally, the optimal microwave power, the optimal ratio of water to raw material, and the optimal microwave extraction time were determined to be 480 W, 50 mL/g, and 8 min according to the single factor experimental design, respectively. Based on the results of single factor experiments, a Box–Behnken experimental design (BBD) with seventeen runs was further applied to optimize the MAE conditions. Table 1 summarized the BBD matrix and the experimental data for the MAE method. By applying multiple regression analysis, a final second-order polynomial equation in terms of coded values was obtained as follows,

(1) $$Y_{1}=1.97+0.047X_{11}+0.062X_{12}-0.11X_{13}-0.040X_{11}X_{12}-0.063X_{11}X_{13}-0.094X_{12}X_{13}-0.20X_{11}^{2}-0.17X_{12}^{2}-0.28X_{13}^{2}$$

where Y_1_ represents the extraction yield; X_11_, X_12_, and X_13_ are microwave power, ratio of water to raw material, and microwave extraction time, respectively.

In the BBD analysis, the F-values and *p*-value were used to check the significance of each coefficient; the greater the F-values and the smaller the P-values were, the more significant the corresponding coefficient [9]. The analysis of variance (ANOVA) was performed to evaluate the predictive model and the variables. As shown in Table 2, the quadratic regression model for the MAE method has a high F-value (44.36) and a very low *p*-value (*p* < 0.0001), indicating that the fitness of the model was extremely significant [17]. Furthermore, the lack of fit for the F-value of 3.55 and *p*-value of 0.1265 (*p* > 0.05) implied the model equation was adequate for predicting the yield of KPS extracted by MAE [18]. Besides, the low value of the coefficient variation (C.V., 2.80%) and the high value of the adeq. precision (16.66) indicated that this model had good precision and reliability [10]. Furthermore, the R^2^ and R_adj_^2^ were 0.9828 and 0.9606, respectively, which revealed that this polynomial model had adequate accuracy and general applicability [19]. In addition, the linear coefficients (X_11_, X_12_, X_13_), interaction coefficients (X_11_X_13_ and X_12_X_13_), and quadratic term coefficients (X_11_^2^, X_12_^2^, and X_13_^2^) were significant (*p* < 0.05), while the interaction coefficient (X_11_X_12_) had no significant influence (*p* > 0.05) on the extraction yield.

**Figure 2:**
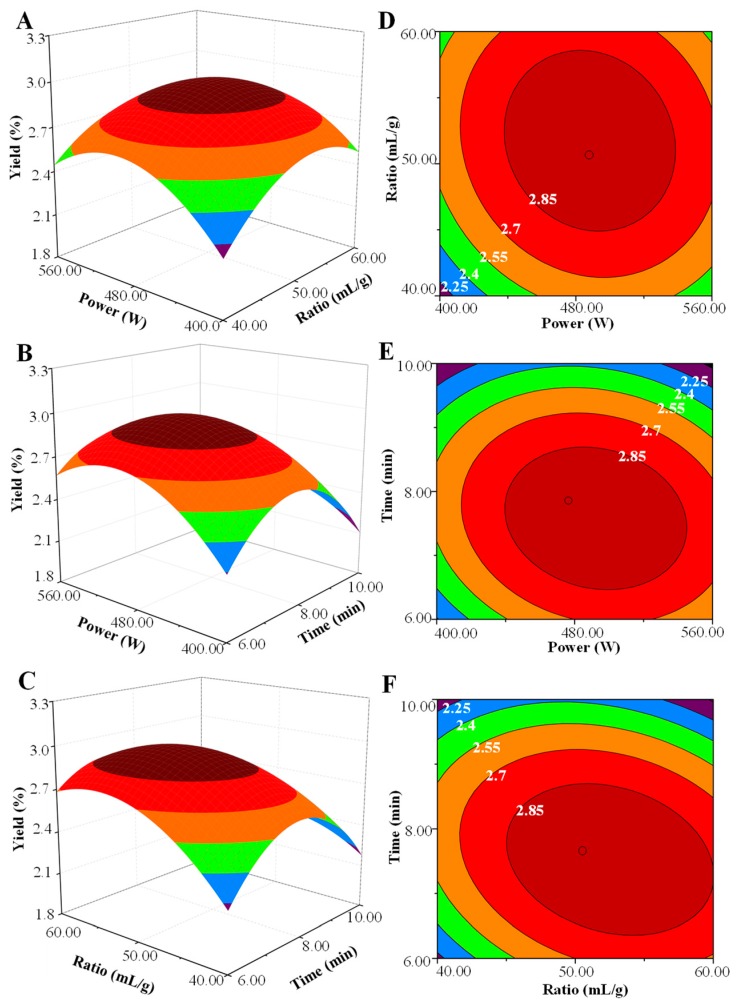
Three-dimensional response surface (**left**) and two-dimensional contour (**right**) plots of microwave-assisted extraction. (**A**,**D**) microwave power and ratio of water to raw material; (**B**,**E**) microwave power and microwave extraction time; (**C**,**F**) ratio of water to raw material and microwave extraction time.

The predicted models were presented in three-dimensional (3D) response surface plots and two-dimensional contour plots as shown in Figure 2. Generally, the response surface with a circular contour plot indicates that the interaction between the corresponding variables is negligible, whereas an elliptical contour plot indicates that the interaction between the corresponding variables is significant [9,11]. In this study, it was clear that the interaction between microwave power and the ratio of water to raw material was not significant. However, the interactions between microwave power and extraction time, and the ratio of water to raw material and extraction time were significant. Furthermore, the model predicted the maximum extraction yield (2.89%) could be obtained under the following optimal extraction conditions: microwave power, 443.2 W, ratio of water to raw material, 52.9 mL/g; microwave extraction time, 7.6 min. However, considering the practical operability, the operating conditions were modified as follows: microwave power, 440 W; ratio of liquid to material, 53.0 mL/g; extraction time, 8.0 min. The actual extraction yield of 2.92 ± 0.08% (n = 3) was obtained, which was in accordance with the predicted value. Results indicated that the model for MAE was accurate and adequate in the present study.

#### 2.1.2. Ultrasonic-Assisted Extraction of KPS

Generally, the factors of ultrasonic power, extraction time, and ratio of water to raw material affect the yield of polysaccharides extracted by UAE [20]. The effects of the ultrasonic amplitude (power), ratio of water to raw material, and ultrasonic extraction time on the yield of KPS are shown in Figure 1, respectively. Briefly, the extraction yield of KPS increased with the increase of ultrasonic amplitude from 25% to 70%, and reached a maximum yield at 70% (Figure 1D). However, above 70%, there was a significant decrease in the yield of KPS, which might be attributed to the degradation of KPS under excessive ultrasonic power [21]. In addition, the yield of KPS increased in the range of the ratio of water to raw material from 20 to 30 mL/g. However, above 30 mL/g, the yield decreased significantly (Figure 1E), which may be because the ultrasound energy in contact with the unit volume would decrease when the ratio of water to raw material increases significantly [20]. The extraction yield of KPS also increased with the increase of extraction time from 2 to 10 min, and reached a maximum yield at 10 min (Figure 1F). However, when the extraction time continued to increase from 10 to 20 min, no significant difference in the extraction yield was observed. Finally, results showed that the optimal ultrasonic amplitude, the optimal ratio of water to raw material, and the optimal ultrasonic extraction time were determined to be 70%, 30 mL/g, and 10 min, according to the single factor experimental design, respectively.

Based on the results of single factor experiments, a BBD with seventeen runs was also applied to optimize the UAE conditions. Table 1 summarized the BBD matrix and the experimental data for the UAE method. By applying multiple regression analysis, a final second-order polynomial equation in terms of coded values was obtained as follows,

(2) $$Y_{2}=1.83-0.053X_{21}+0.032X_{22}+0.12X_{23}+0.023X_{21}X_{22}-0.11X_{21}X_{23}+0.016X_{22}X_{23}-0.26X_{21}^{2}-0.29X_{22}^{2}-0.18X_{23}^{2}$$

where Y_2_ represents the extraction yield; X_21_, X_22_, and X_23_ are ultrasonic amplitude, ratio of water to raw material, and ultrasonic extraction time, respectively.

As shown in Table 2, the quadratic regression model for the UAE method has a high F-value (37.34) and a very low *p*-value (*p* < 0.0001), indicating that the fitness of the model was extremely significant [17]. Furthermore, the lack of fit for the F-value of 3.46 and *p*-value of 0.1308 (*p* > 0.05) implied the model equation was adequate for predicting the yield of KPS extracted by UAE [18]. Besides, the low value of the coefficient variation (C.V., 3.79%) and the high value of the adeq. precision (15.25) indicated that this model had good precision and reliability [10]. The coefficient of determination (R^2^) and adjusted coefficient of determination (R_adj_^2^) were 0.9796 and 0.9534, respectively, which showed a good agreement between the experimental and the predicted values of the yield with the goodness-of-fit of the regression equation [22]. In addition, the linear coefficients (X_21_, X_23_), interaction coefficient (X_21_X_23_), and quadratic term coefficients (X_21_^2^, X_22_^2^, and X_23_^2^) were significant (*p* < 0.05), while the linear coefficient (X_22_), the interaction coefficients (X_21_X_22_ and X_22_X_23_) had no significant influence (*p* > 0.05) on the extraction yield.

**Figure 3:**
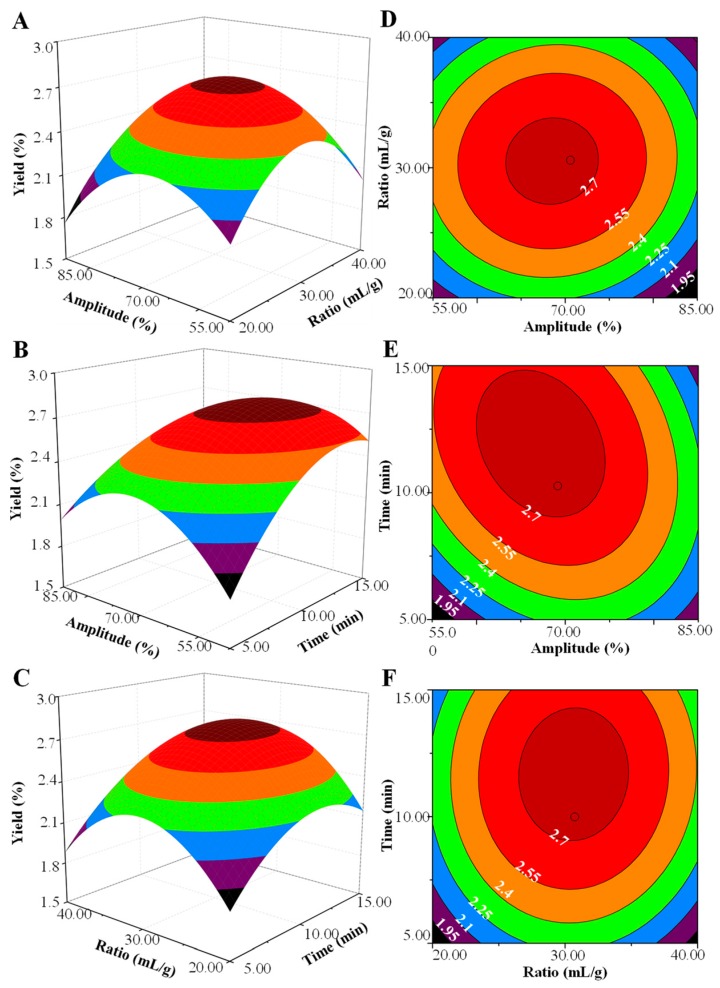
Three-dimensional response surface (**left**) and two-dimensional contour (**right**) plots of ultrasonic-assisted extraction. (**A**,**D**) ultrasonic amplitude and ratio of water to raw material; (**B**,**E**) ultrasonic amplitude and ultrasonic extraction time; (**C**,**F**) ratio of water to raw material and ultrasonic extraction time.

Moreover, the 3D response surface and 2D contour plots of UAE are shown in Figure 3. The results showed that the interaction between the ultrasonic amplitude and extraction time was significant. However, the interaction between ultrasonic amplitude and ratio of water to raw material, and the extraction time and ratio of water to raw material were not significant. Furthermore, the model predicted that the maximum extraction yield (2.84%) could be obtained under the following optimal extraction conditions: ultrasonic amplitude, 67.3%; ratio of water to raw material, 30.6 mL/g; ultrasonic extraction time, 12.0 min. Considering the operability in the actual processing procedure, the verification experiment was carried out under the following conditions: ultrasonic amplitude of 68.0%, ratio of water to raw material of 31.0 mL/g, and ultrasonic extraction time of 12.0 min. Under these optimal UAE conditions, the actual extraction yield of KPS was 2.82 ± 0.10% (n = 3), which was in good agreement with the predicted value. Results indicated that the model for UAE was accurate and adequate in the present study.

### 2.2. Preliminary Characterization of KPS

#### 2.2.1. Chemical Composition of KPS

As shown in Table 3, the extraction yields of KPS extracted by the MAE method (KPS-M), KPS extracted by the UAE method (KPS-U), and KPS extracted by HWE method (KPS-H) were determined to be 2.92 ± 0.08%, 2.82 ± 0.10%, and 2.86 ± 0.13%, respectively. Results showed that the MAE, UAE, and HWE had no significant effects on the extraction yields of KPS under their optimal extraction conditions as previously mentioned. However, considering the extraction time and extraction temperature of MAE, UAE, and HWE, both UAE and MAE could be better than HWE [13,15]. High carbohydrate contents and low protein contents were observed in KPS-M (78.21 ± 1.42% and 3.50 ± 0.12%, respectively), KPS-U (74.80 ± 1.60% and 6.08 ± 0.15%, respectively), and KPS-H (76.18 ± 1.46% and 4.28 ± 0.09%), respectively, which indicated that the polysaccharides were the major biological components in KPS obtained by different extraction methods. In addition, total uronic acid content in KPS-H (45.7 ± 0.98%) was significantly (*p* < 0.05) higher than that in KPS-M (43.88 ± 0.65%) and KPS-U (43.32 ± 0.73%). The results suggested that different extraction methods had significant effects on the contents of uronic acid in polysaccharides [13,14]. Indeed, the lower content of uronic acids in KPS-M and KPS-U might be attributed to the degradation of KPS under microwave and ultrasonic treatment.

#### 2.2.2. Molecular Weights and Constituent Monosaccharides of KPS

**Figure 4:**
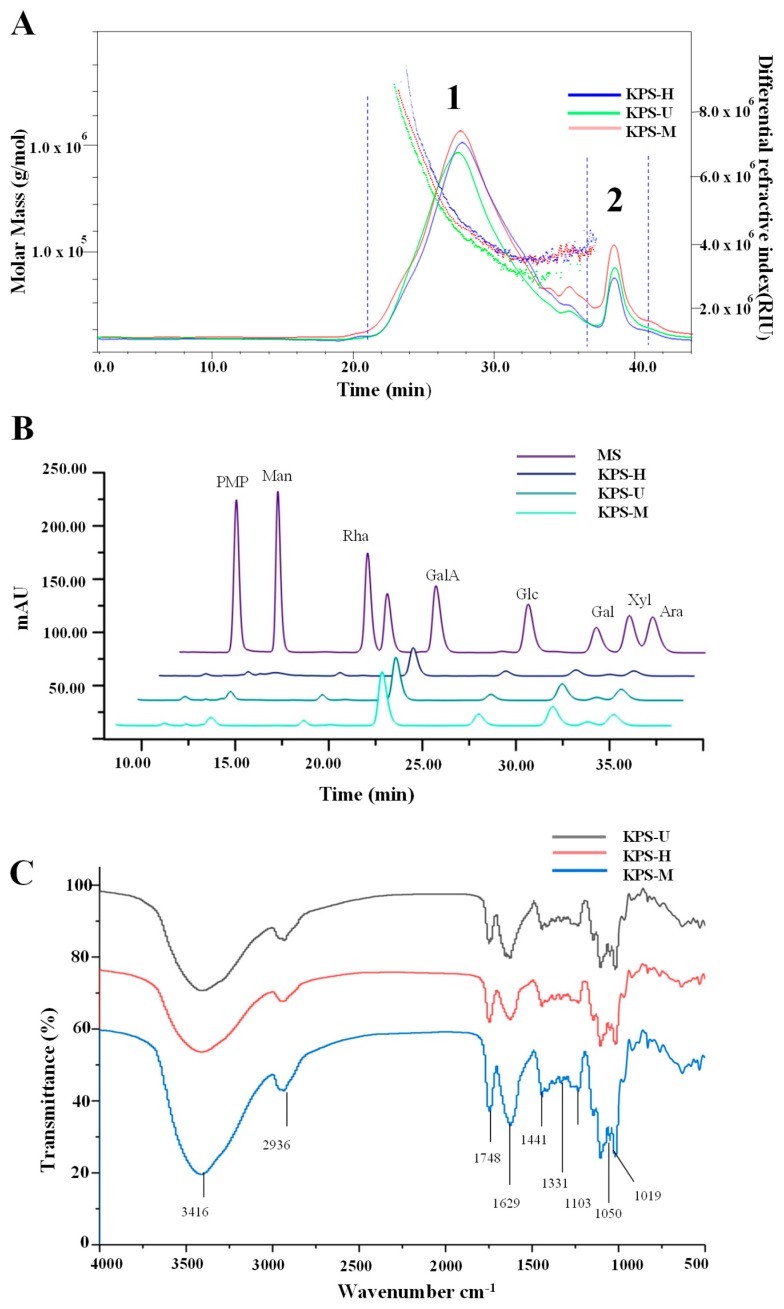
HPSEC chromatograms (**A**), HPLC profiles (**B**), and FT-IR spectra (**C**) of KPS-M, KPS-U, and KPS-H. KPS-M, kiwifruit polysaccharides (KPS) extracted by microwave-assisted extraction; KPS-U, KPS extracted by ultrasound-assisted extraction; KPS-H, KPS extracted by hot water extraction; MS, mixed standard of monosaccharides; Man, Mannose; Rha, Rhamnose; GlcA, Glucuronic acid; GalA, Galacturonic acid; Glc, Glucose; Gal, Galactose; Xyl, Xylose; Ara, Arabinose.

Generally, the bioactivities of natural polysaccharides are associated with their molecular weights and constituent monosaccharides [23]. Figure 4A showed the HPSEC-RID chromatograms of KPS-M, KPS-U, and KPS-H. Two polysaccharide fractions (Figure 4A, fraction 1 and 2) were detected in KPS-M, KPS-U, and KPS-H. The molecular weight of fraction 2 could not be precisely determined due to the relatively poor resolution of the column and the co-elution of various different molecules from 38 min to 42 min. As shown in Table 3, the molecular weights of polysaccharide fraction 1 in KPS ranged from 1.689 × 10^5^ Da to 1.955 × 10^5^ Da, which were much lower than that of polysaccharides isolated from the skin and seed of gold kiwifruit [24]. Results showed that the molecular weights of KPS-H extracted by HWE were significantly (*p* < 0.05) higher than that of KPS-U extracted by UAE and KPS-M extracted by MAE. Results suggested that both the UAE and MAE methods could degrade the molecular weights of KPS. Similar studies have shown that the molecular weights of polysaccharides extracted by UAE and MAE are lower than that of conventional HWE [13,14,25]. Furthermore, the polydispersities of polysaccharide fraction 1 in KPS-M, KPS-U, and KPS-H were determined to be 1.827, 1.724, and 1.833, respectively.

Furthermore, Figure 4B showed that the HPLC-UV profiles of KPS-M, KPS-U, and KPS-H were similar. Results showed that constituent monosaccharides in KPS-M, KPS-U, and KPS-H were similar, which were measured as Man, Rha, GalA, Glc, Gal, Xyl, and Ara. The major monosaccharide compositions were similar to some previous studies, suggesting the polysaccharides obtained in this study are pectic polysaccharides [24]. As shown in Table 3, the molar rations of Man, Rha, GalA, Glc, Gal, Xyl, and Ara in KPS-M, KPS-U, and KPS-H were different, which were determined to be about 0.36:0.23:3.28:1.00:1.69:0.24:0.93, 0.77:0.47:5.16:1.00:2.91:0.36:1.86, and 0.25:0.35:4.07:1.00:1.24:0.24:1.01, respectively. The results suggested that the UAE and MAE methods had no effect on the types of constituent monosaccharides in KPS, but significantly affected their molar ratios. The possible reason might be attributed to the degradation of molecular weights and uronic acids of KPS under microwave and ultrasonic treatment. Similar studies have shown that different extraction techniques affect the molar ratios of compositional monosaccharides of polysaccharides [13,15].

#### 2.2.3. FT-IR Spectra and Degree of Esterification of KPS

The FT-IR spectra of KPS-M, KPS-U, and KPS-H were recorded in the range of 4000 to 400 cm^−1^, and the results are shown in Figure 4C. No obvious differences were observed among the FT-IR spectra of different KPS. The FT-IR spectrum of KPS showed a strong absorbance band at 3416 cm^−1^, which represented the stretching vibration of the hydroxyl group in the constituent sugar residues [26]. The weak peak at 2936 cm^−1^ was assigned to the stretching vibration of C–H while the absorption bands at 1748 cm^−1^ represented the asymmetric stretching vibration of carbonyl double-bond (C=O). Furthermore, the intense peak appeared at 1629 cm^−1^ was the C=O asymmetric stretching of −COO, suggesting the existence of uronic acid [27,28]. In addition, the bands at 1441 and 1331 cm^−1^ were assigned to C–O stretching vibrations and O–H deformation vibrations [20]. The strong absorption band between 1200–1000 cm^−1^ at 1103, 1050, and 1019 cm^−1^ were assigned to the stretching vibration of C–O–C and C–O–H bonds [29]. The degree of esterification (DE) of KPS-U, KPS-M and KPS-H were also investigated by FT-IR spectroscopy analysis. According to the absorbance at 1748 cm^−1^ (considered as esterified uronic acids) and 1629 cm^−1^ (considered as free uronic acids), the significantly (*p* < 0.05) highest DE values (55.02%) were observed in KPS-U, followed by the lower values (48.38%) in KPS-U, and lowest values (43.33%) in KPS-M. Results suggested that the MAE and UAE methods significantly affected the DE of KPS. Previous studies have also indicated that the low DE is observed in pectins extracted under harsh extraction conditions (such as high temperature, high microwave power, and long microwave extraction time) [30,31], because these harsh conditions could increase de-esterification of polygalacturonic chains.

### 2.3. Antioxidant Activities of KPS

**Figure 5:**
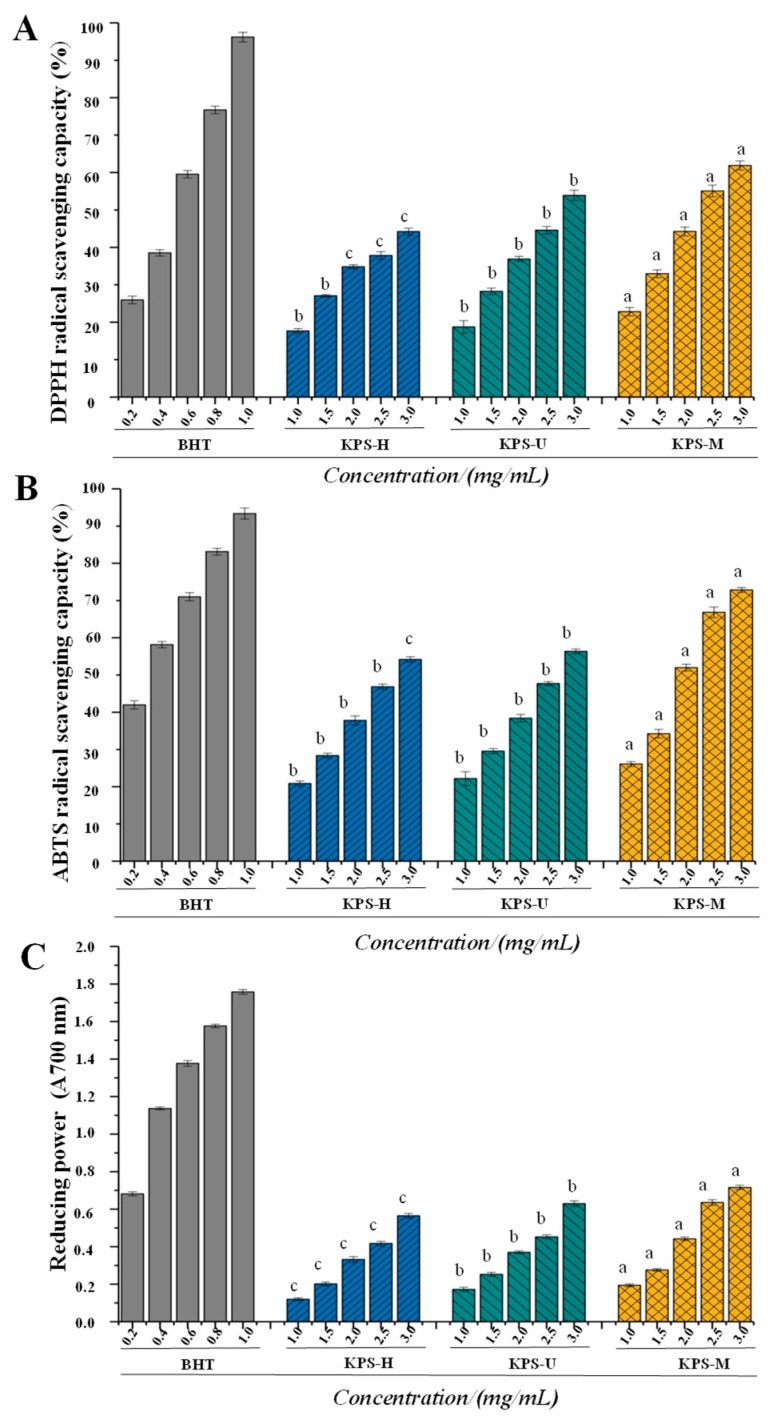
DPPH radical scavenging activity (**A**), ABTS radical cation scavenging activity (**B**), and reducing power (**C**) of KPS-M, KPS-U, and KPS-H. KPS-M, kiwifruit polysaccharides with microwave-assisted extraction; KPS-U, kiwifruit polysaccharides with ultrasonic-assisted extraction; KPS-H, kiwifruit polysaccharides with hot water extraction; BHT, butylated hydroxytoluene; The error bars are standard deviations; Significant (*p* < 0.05) differences are shown by data bearing different letters (a–c); Statistical significances were carried out by ANOVA and Ducan’s test.

Pharmacological studies have shown that kiwifruit possesses strong antioxidant activity [4]. However, the antioxidant activities of polysaccharides in kiwifruit have seldom been investigated. The DPPH radical scavenging activities, ABTS radical scavenging activities, and reducing power of KPS-M, KPS-U, and KPS-H are shown in Figure 5. Briefly, as shown in Figure 5A, the DPPH radical scavenging activities of KPS exhibited a dose-dependent manner. The results showed that the significantly (*p* < 0.05) highest DPPH radical scavenging activities were observed in KPS-M at different concentrations of 1 to 3 mg/mL, followed by lower DPPH radical scavenging activities in KPS-U, and the lowest DPPH radical scavenging activities in KPS-H. The results suggested that the KPS-M extracted by the MAE method exhibited stronger antioxidant activities than that of KPS-U extracted by the UAE method and KPS-H extracted by HWE method. The MAE method could be a good potential technique for the extraction of kiwifruit polysaccharides with high antioxidant activities. In addition, the IC_50_ values of DPPH radical scavenging capacities of KPS-M, KPS-U, and KPS-H were determined as 2.33 mg/mL, 2.78 mg/mL, and 3.38 mg/mL, respectively. It shows that KPS-M has a strong antioxidant activity, even if the IC_50_ values of DPPH radical scavenging capacities of it is higher than butylated hydroxytoluene (BHT) (a positive control, IC_50_ = 1.23 mg/mL). Therefore, the result indicated that KPS-M had a more powerful ability to transfer electron or hydrogen atom to DPPH than KPS-U and KPS-H. Furthermore, as shown in Figure 5B, the ABTS radical scavenging activities of KPS also exhibited a dose-dependent manner. Results showed that the significantly (*p* < 0.05) highest ABTS radical scavenging activities were also observed in KPS-M at different concentrations of 1 to 3 mg/mL, followed by lower antioxidant activities in KPS-U and KPS-H. Indeed, the IC_50_ values of ABTS radical scavenging activities of KPS-M, KPS-U, and KPS-H were determined to be 1.98 mg/mL, 2.64 mg/mL, and 2.73 mg/mL, respectively. Compared with the BHT (IC_50_ = 0.29 mg/mL), KPS-M also exhibited strong ABTS radical scavenging activities. The results further confirmed that KPS-M exhibited the strongest antioxidant activities among KPS extracted by different methods. As shown in Figure 5C, the significantly (*p* < 0.05) highest reducing power was also observed in KPS-M at different concentrations of 1 to 3 mg/mL, followed by lower reducing power in KPS-U, and the lowest reducing power in KPS-H. Although the reducing power of KPS-M was lower than that of BHT, its absorbance at 700 nm still reached 0.71 at the concentration of 3.0 mg/mL. All results suggested that KPS could be one of the major contributors toward the antioxidant activities of kiwifruit. Generally, the antioxidant activities of natural polysaccharides are correlated to their structure features, molecular weights, and compositional monosaccharides (uronic acids) [14,32,33]. It is estimated that the presence of electrophilic groups like keto or aldehyde in acidic polysaccharides facilitates the liberation of hydrogen from O–H bond, and these groups can improve the radical scavenging activities [34]. In the present study, the high antioxidant activities (DPPH radical scavenging activities, ABTS radical scavenging activities, and reducing power) observed in KPS-M might be partially attributed to its low molecular weight and high content of unmethylated galacturonic acids as previously mentioned [35,36,37]. However, the further purification, structural characterization, and in vitro and in vivo antioxidant activities of KPS are required to reveal their structure-bioactivity relationships.

## 3. Materials and Methods

### 3.1. Material and Chemicals

Kiwifruit (*Actinidia chinensis* Planch. cv. Hongshi) were harvested at a commercial orchard located in Mianzhu, Sichuan Province, China, on 28 September 2017. All samples were harvested at their commercial maturity, and then they were ripened at room temperature (20–25 °C) to reach an average firmness of 0.5–0.7 kg/cm^2^. The average fruit weight, soluble solids content, and dry matter of ripened kiwifruit samples were 58.07 g, 16.3%, and 16.49%, respectively. Subsequently, the samples were peeled, sliced, freeze-dried, crushed into powder, and sieved using a 60-mesh screen. The resulting powdered samples were stored at −20 °C for further analysis.

Trifluoroacetic acid, rhamnose (Rha), mannose (Man), glucuronic acid (GlcA), galacturonic acid (GalA), glucose (Glc), galactose (Gal), xylose (Xyl), arabinose (Ara), 1-phenyl-3-methyl-5-pyrazolone (PMP), 2,2-diphenyl-1-(2,4,6-trinitrophenyl) hydrazyl (DPPH), 2,2′-azino-bis(3-ethylbenzothiazoline-6-sulphonic acid) (ABTS), and butylated hydroxytoluene (BHT), were purchased from Sigma-Aldrich (St. Louis, MO, USA). All other reagents and chemicals used were of analytical grade.

### 3.2. Extraction of Polysaccharides from Kiwifruit

#### 3.2.1. Microwave-Assisted Extraction of KPS

Both single factor experimental design and Box–Behnken experimental design were used for the optimization of microwave-assisted extraction (MAE) conditions. Briefly, the kiwifruit powder (1.0 g) was firstly refluxed with 10 mL of 80% (*v*/*v*) ethanol at 80 °C for 2 h to remove most of the small molecules. Subsequently, the extract residue was extracted with deionized water by MAE (MKJ-J1-3, Qingdao Makewave Microwave Applied Technology Co., Ltd., Qingdao, Shandong, China), and the effects of microwave power (240, 320, 400, 480, and 560 W), ratio of water to raw material (20, 30, 40, 50, and 60 mL/g) and extraction time (2, 4, 6, 8, and 10 min) on the yield of the KPS were investigated using a single factor experimental design. After centrifugation at 4000× *g* for 15 min (Heraeus Multifuge X3R Centrifuge, Thermo Fisher Scientific, Waltham, MA, USA), the supernatants were concentrated to 1/3 of the origin volume by a rotary evaporator (RE-52AA, Yarong Company, Shanghai, China) under a vacuum at 60 °C. Then, the starch of mixture was removed by the heat-stable α-amylase at 80 °C. The enzymes were inactivated at 95 °C for 20 min, and the mixture was centrifuged at 4000× *g* for 20 min. Furthermore, the supernatants were precipitated with four volumes of 95% ethanol (*v*/*v*) overnight at 4 °C. The precipitations were washed with 70% ethanol (*v*/*v*) three times, and then dissolved in deionized water. Finally, the crude kiwifruit polysaccharides (KPS-W) were freeze dried, and stored at −20 °C for further analysis.

Furthermore, based on the results of the single factor experiments, a three-level Box–Behnken experimental design (BBD) with three factors was applied to optimize the MAE conditions. The microwave power (X_11_, W), ratio of water to raw material (X_12_, mL/g), and microwave extraction time (X_13_, min) were preferred for independent variables. According to the BBD design, 17 experimental runs with 1 block and 5 center points were performed. The variables and their levels, with both coded and actual values were presented in Table 1. The obtained data were analyzed by the statistical of the Design Expert software 8.0.5 (Stat-Ease Inc., Minneapolis, MN, USA). The significance of the model was evaluated by analysis of variance (ANOVA). Experimental data from BBD were explained by the second-order polynomial model as follows [20],

(3) $$Y=A_{0}+\overset{3}{\underset{i=1}{\sum }}A_{i}X_{i}+\overset{3}{\underset{i=1}{\sum }}A_{\mathrm{ii}}{X_{i}}^{2}+\overset{2}{\underset{i=1}{\sum }}\overset{3}{\underset{j=i+1}{\sum }}A_{\mathrm{ij}}X_{i}X_{j}$$

where Y is the predicted response; X_i_ and X_j_ are different variables (i ≠ j); A_0_, A_i_, A_ii_, and A_ij_, are regression coefficients for intercept, linearity, square, and interaction, respectively.

#### 3.2.2. Ultrasonic-Assisted Extraction of KPS

Both single factor experimental design and Box–Behnken experimental design were also used for the optimization of ultrasonic-assisted extraction (UAE) conditions. The KPS were extracted by ultrasonic-assisted extraction with an Ultrasonic Processor (650 W, 24 kHz, Scientz Company, Ningbo, China) at room temperature. Briefly, the kiwifruit powder (1.0 g) was firstly refluxed with 10 mL of 80% (*v*/*v*) ethanol at 80 °C for 2 h to remove most of small molecules. Subsequently, the extract residue was extracted with deionized water by UAE, and the effects of ultrasonic amplitude (25%, 40%, 55%, 70% and 85%), ratio of water to raw material (20, 30, 40, 50 and 60 mL/g) and ultrasonic extraction time (2, 5, 10, 15, and 20 min) on the yield of the KPS were investigated using a single factor experimental design. Finally, the kiwifruit polysaccharides (KPS-U) were obtained according to the same treatment processes as described in Section 3.2.1.

Furthermore, based on the results of the single factor experiments, a three-level Box–Behnken experimental design (BBD) with three factors was also applied to optimize the UAE conditions. The ultrasonic amplitude (X_21_, %), ratio of water to raw material (X_22_, mL/g), and ultrasonic extraction time (X_23_, min) were preferred for independent variables. The variables and their levels, with both coded and actual values are also presented in Table 1. Statistical analysis was performed as described in Section 3.2.1.

#### 3.2.3. Hot Water Extraction of KPS

Hot water extraction of KPS was performed according to a previously reported method with slight modifications [10]. Briefly, the kiwifruit (1.0 g) were firstly refluxed with 10 mL of 80% (*v*/*v*) ethanol at 80 °C for 2 h to remove most of small molecules. Subsequently, the kiwifruit polysaccharides (KPS) were extracted twice with 30 mL of deionized water at 90 °C for 2 h. Finally, the kiwifruit polysaccharides (KPS-H) were obtained according to the same treatment processes as described in Section 3.2.1.

### 3.3. Characterization of KPS

#### 3.3.1. Physicochemical Properties Analysis

The content of total polysaccharides in KPS was determined by the phenol-sulfuric acid method with a mixture standard [38]. The mixture standard was prepared by 50% of GalA, 30% of Ara, and 20% of Gal. The content of uronic acids in KPS was determined by using the m-hydroxydiphenyl method with GalA as a standard [39]. The content of proteins in KPS was determined by using Bradford’s method with bovine serum albumin as a standard [40].

#### 3.3.2. Determination of Molecular Weights

The absolute molecular weights (M_w_) and polydispersities (M_w_/M_n_) were measured by high-performance size exclusion chromatography coupled with multi-angle laser light scattering and refractive index detector (HPSEC-MALLS-RID) according to our previously reported method with minor modifications [41]. In brief, HPSEC-MALLS-RID measurements were carried out on a multi-angle laser light scattering detector (DAWN HELEOS, Wyatt Technology Co., Santa Barbara, CA, USA) with an Agilent 1260 series LC system (Agilent Technologies, Palo Alto, CA, USA). TSK-Gel G5000PWXL (300 mm × 7.8 mm, i.d.) and TSK-Gel G3000PWXL (300 mm × 7.8 mm, i.d.) were used in series at 30 °C. The MALLS instrument was equipped with a He-Ne laser (λ = 658 nm). An Optilab rEX refractometer (DAWN EOS, Wyatt Technology Co., Santa Barbara, CA, USA) was simultaneously connected. The mobile phase was 0.9% NaCl aqueous solution at a flow rate of 0.5 mL/min. The sample concentration was about 1.0 mg/mL. An injection volume of 100 μL was used. The dn/dc value of KPS was selected as 0.15 mL/g according to a previous study [41].

#### 3.3.3. Determination of Constituent Monosaccharides

Constituent monosaccharides of KPS-M, KPS-U, and KPS-W were measured by high-performance liquid chromatography (HPLC) analysis according to a previously reported method with some modifications [42]. Briefly, each sample (4.0 mg) was hydrolyzed with 2.0 M trifluoroacetic acid (2.0 mL) at 95 °C for 10 h. After hydrolysis, the hydrolysates were evaporated to dryness by a rotary evaporator under a vacuum, and washed with methanol three times to remove the residue of trifluoroacetic acid. Subsequently, the dried hydrolyzates were dissolved in 1 mL of water for subsequent derivatization.

Furthermore, 50 µL of hydrolyzates were mixed with 50 µL of 0.6 M sodium hydroxide and 100 µL of 0.5 M PMP methanol solution. The mixture was incubated at 70 °C for 100 min with continuous shaking. Then, 80 µL of 0.3 M hydrochloric acid solution was used to neutralize the mixture, and the mixture was diluted to 1 mL with pure water. 1 mL of chloroform was added. After vigorous shaking and layering, the organic phase was discarded. The operation was performed in triplicate, and finally the solution was passed through a 0.22 µm organic syringe filter for HPLC analysis. A standard solution, containing Rha, Man, GlcA, GalA, Glc, Gal, Xyl, and Ara, was derivatized as described above. Finally, The PMP derivatives were analyzed by a Dionex UltiMate 3000 HPLC system (ThermoFisher Scientific, Waltham, MA, USA) with a ZORBAX Eclipse XDB-C18 column (4.6 × 250 mm i.d. 5 µm, Agilent Technologies Inc., CA, USA) and a diode array detector (DAD, ThermoFisher Scientific). A 20 µL of PMP derivative was injected into the HPLC system at the operation temperature of 30 °C, and eluted with a mixture of 0.1 M phosphate buffer solution (pH = 6.7) and acetonitrile (83: 17, *v*/*v*) at a flow rate of 1.0 mL/min. The wavelength of DAD was set at 245 nm.

#### 3.3.4. Fourier Transform Infrared (FT-IR) Spectroscopy Analysis

Each sample (1.0 mg) was mixed with 100 mg of dried KBr, and pressed into a disk for the analysis. The IR spectra were recorded in the frequency range of 4000–400 cm^−1^ with a Nicolet iS 10 FT-IR (ThermoFisher Scientific). Furthermore, the esterification degree (DE) of KPS-M, KPS-U, and KPS-H was determined from FT-IR spectra according to previously reported methods [43,44]. The determination of DE was based on the band areas at 1700–1750 cm^−1^ (esterified uronic acids) and 1600–1630 cm^−1^ (free uronic acids). DE was calculated according to the equation as follows,

(4) $$\mathrm{DE}\left(\%\right)=\left(\frac{A_{1748}}{A_{1748}+A_{1629}}\right)\times 100\%$$

### 3.4. Evaluation of Antioxidant Activities of Polysaccharides from Kiwifruit

#### 3.4.1. DPPH Radical Scavenging Activity

The DPPH radical scavenging activity of KPS-M, KPS-U, and KPS-H was determined according to our previously reported method with minor modifications [45]. Briefly, 200 µL of 0.35 mM DPPH solution was mixed with 20 µL of each sample at different concentrations (1, 1.5, 2, 2.5, and 3 mg/mL) or deionized water as a negative control in a 96-well microplate. Then the mixture was incubated at 37 °C for 30 min in dark, and the absorbance was measured at 517 nm. BHT was used as a positive control. The DPPH radical scavenging activity (%) was calculated as follows,

(5) $$\mathrm{DPPH}\;\mathrm{radical}\;\mathrm{scavenging}\;\mathrm{activity}\;\left(\%\right)=\left(1-\frac{A_{\mathrm{sample}}-A_{\mathrm{control}}}{A_{\mathrm{blank}}}\right)\times 100\%$$

where A_sample_ is the absorbance of the mixture of sample and DPPH work solution; A_control_ is the absorbance of the mixture of deionized water and sample; A_blank_ is the absorbance of the mixture of deionized water and DPPH work solution.

#### 3.4.2. ABTS Radical Cation Scavenging Activity

The ABTS radical cation scavenging activity of KPS-M, KPS-U, and KPS-H was measured according to our previously reported method with minor modification [45]. Briefly, the ABTS radical cation solution was generated by the interaction of 7 mM ABTS solution and 2.45 mM aqueous potassium persulfate at room temperature for at least 16 h in dark. The ABTS radical cation solution was diluted with phosphate buffer (0.2 M, pH 7.4) to an absorbance of 0.750 ± 0.02 at 734 nm. Then, 200 μL of ABTS radical cation working solution was mixed with 20 μL of each sample at different concentrations (1, 1.5, 2, 2.5, and 3 mg/m) or phosphate buffer as a negative control in a 96-well microplate to react at 30 °C for 20 min. The absorbance at 734 nm was measured. BHT was used as a positive control, and the ABTS radical scavenging activity was calculated as follows,

(6) $$\mathrm{ABTS}\;\mathrm{radical}\;\mathrm{scavenging}\;\mathrm{activity}\;\left(\%\right)=\left(1-\frac{A_{\mathrm{sample}}-A_{\mathrm{control}}}{A_{\mathrm{blank}}}\right)\times 100\%$$

where A_sample_ is the absorbance of the mixture of sample and ABTS work solution; A_control_ is the absorbance of the mixture of deionized water and sample; A_blank_ is the absorbance of the mixture of deionized water and ABTS work solution.

#### 3.4.3. Reducing Power

The reducing power was determined according to our previously reported method with minor modifications [45]. Briefly, an aliquot of 100 µL each sample at different concentrations (1, 1.5, 2, 2.5, and 3 mg/mL) was mixed with 100 µL of potassium ferricyanide (1%, *w*/*v*) in phosphate buffer (pH 6.8, 20 mM). After the mixture was incubated at 50 °C for 20 min, 100 µL of trichloroacetic acid (10%, *w*/*v*) was added, followed by centrifugation at 3000× *g* for 10 min. The supernatant (100 µL) was mixed with 100 µL of distilled water and 20 µL of ferric chloride (0.1%, *w*/*v*). The absorbance was measured at 700 nm after 30 min incubation. The blank control contained all reagents except the sample. BHT was used as the standard, and the reducing power of kiwifruit polysaccharide was expressed as absorbance at 700 nm.

### 3.5. Statistical Analysis

All experiments were conducted in triplicate, and data were expressed in means ± standard deviations. Statistical analysis was performed using Origin 9.0 software (OriginLab Corporation, Northampton, MA, USA). Statistical significances were carried out by one-way analysis of variance (ANOVA), followed by Duncan’s test. Values of *p* < 0.05 were considered as statistically significant.

## 4. Conclusions

In this study, the optimal extraction conditions of MAE and UAE for the extraction of KPS were obtained by using response surface methodology. Furthermore, different extraction techniques significantly affected the contents of uronic acids, molecular weights, molar ratio of constituent monosaccharides, and degree of esterification of KPS. Furthermore, KPS exhibited strong antioxidant activities. The high antioxidant activities observed in KPS-M extracted by the MAE method might be partially attributed to its low molecular weight and high content of unmethylated galacturonic acid. Results suggested that the MAE method could be a good potential technique for the extraction of KPS with high antioxidant activity, and KPS could be further explored as functional food ingredients.

## Figures and Tables

**Table 1:** Box–Behnken design with independent variables and observed values for microwave-assisted extraction (MAE) and ultrasonic-assisted extraction (UAE).

Runs	Levels of Independent Factors (MAE) ^a^			Extraction Yield %	Levels of Independent Factors (UAE) ^b^			Extraction Yield %
	X_11_ (W)	X_12_ (min)	X_13_ (mL/g)		X_21_ (%)	X_22_ (mL/g)	X_23_ (min)	
1	1(560)	0(50)	1(10)	2.10	0(70)	1(40)	−1(5)	1.80
2	1(560)	−1(40)	0(8)	2.46	−1(55)	0(30)	1(15)	2.49
3	0(480)	1(60)	1(10)	2.04	1(85)	1(40)	0(10)	1.98
4	−1(400)	−1(40)	0(8)	2.19	1(85)	−1(20)	0(10)	1.76
5	1(560)	1(60)	0(8)	2.51	0(70)	0(30)	0(10)	2.60
6	0(480)	−1(40)	1(10)	2.10	−1(55)	0(30)	−1(5)	1.89
7	−1(400)	1(60)	0(8)	2.46	0(70)	−1(20)	1(15)	2.22
8	0(480)	0(50)	0(8)	3.00	−1(55)	−1(20)	0(10)	1.92
9	0(480)	0(50)	0(8)	2.96	0(70)	0(30)	0(10)	2.75
10	0(480)	0(50)	0(8)	2.87	0(70)	1(40)	1(15)	2.31
11	−1(400)	0(50)	−1(6)	2.19	0(70)	−1(20)	−1(5)	1.82
12	1(560)	0(50)	−1(6)	2.51	0(70)	0(30)	0(10)	2.78
13	0(480)	0(50)	0(8)	2.98	−1(55)	1(40)	0(10)	2.01
14	0(480)	1(60)	−1(6)	2.75	1(85)	0(30)	1(15)	1.94
15	0(480)	0(50)	0(8)	2.96	0(70)	0(30)	0(10)	2.70
16	0(480)	−1(40)	−1(6)	2.25	0(70)	0(30)	0(10)	2.82
17	−1(400)	0(50)	1(10)	2.16	−1(55)	0(30)	−1(5)	2.01

^a^ MAE: X_11_, microwave power (W); X_12_, ratio of water to raw material (mL/g); X_13_, microwave extraction time (min); ^b^ UAE: X_21_, ultrasonic amplitude (%); X_22_, ratio of water to raw material (mL/g); X_23_, ultrasonic extraction time (min).

**Table 2:** Analysis of the variance of the regression equation and coefficients of microwave-assisted extraction (MAE) and ultrasonic-assisted extraction (UAE).

Source ^a^	MAE					UAE				
	Sum of Squares	d*f* ^b^	Mean Square	F-Value	*p*-Value ^c^	Sum of Squares	d*f* ^b^	Mean Square	F-Value	*p*-Value ^c^
Model	0.87	9	0.097	44.36	<0.0001 **	1.06	9	0.12	37.34	<0.0001 **
X_11_ (X_21_)	0.018	1	0.018	8.25	0.0239 *	0.022	1	0.022	7.04	0.0328 *
X_12_ (X_22_)	0.031	1	0.031	14.30	0.0069 **	8.21 × 10^−3^	1	8.21 × 10^−3^	2.59	0.1513
X_13_ (X_23_)	0.092	1	0.092	41.99	0.0003 **	0.12	1	0.12	36.59	0.0005 **
X_11_X_12_ (X_21_X_22_)	6.28 × 10^−3^	1	6.28 × 10^−3^	2.88	0.1336	2.11 × 10^−3^	1	2.11 × 10^−3^	0.66	0.4417
X_11_X_13_ (X_21_X_23_)	0.016	1	0.016	7.18	0.0315 *	0.049	1	0.049	15.46	0.0057 **
X_12_X_13_ (X_22_X_23_)	0.036	1	0.036	16.29	0.0050 **	1.08 × 10^−3^	1	1.08 × 10^−3^	0.34	0.5777
X_11_^2^ (X_21_^2^)	0.16	1	0.16	75.50	<0.0001 **	0.28	1	0.28	89.51	<0.0001 **
X_12_^2^ (X_22_^2^)	0.12	1	0.12	54.39	0.0002 **	0.36	1	0.26	112.54	<0.0001 **
X_13_^2^ (X_23_^2^)	0.32	1	0.32	147.8	<0.0001 **	0.14	1	0.14	43.83	0.0003 **
Residual error	0.013	7	2.18 × 10^−3^			0.02	7	3.17 × 10^−3^		
Lack of fit	0.015	3	3.70 × 10^−3^	3.55	0.1265	0.016	3	5.33 × 10^−3^	3.46	0.1308
Pure error	4.17 × 10^−3^	4	1.04 × 10^−3^			6.16 × 10^−3^	4	1.54 × 10^−3^		
Correlation total	0.89	16				1.09	16			

MAE, R^2^ = 0.9828 R^2^_adj_ = 0.9606, coefficient of variation = 2.80%, adeq. precision = 16.66; UAE, R^2^ = 0.9796, R^2^_adj_ =0.9534, coefficient of variation = 3.79%, adeq. precision = 15.25; ^a^ X_11_, microwave power (W); X_12_, ratio of water to raw material (mL/g); X_13_, microwave extraction time (min); X_21_, ultrasonic amplitude (%); X_22_, ratio of water to raw material (mL/g); X_23_, ultrasonic extraction time (min); ^b^ d*f*, the degree of freedom; ^c^ *, Significantly different (*p* < 0.05), ** Extremely significantly different (*p* < 0.01).

**Table 3:** Chemical composition, molecular weights (M_w_), polydispersity (M_w_/M_n_), and constituent monosaccharides of KPS-M, KPS-U and KPS-H.

	KPS-M	KPS-U	KPS-H
Extraction yield (%)	2.92 ± 0.08 ^a^	2.82 ± 0.10 ^a^	2.86 ± 0.13 ^a^
Total carbohydrate (%)	78.21 ± 1.42 ^a^	74.80 ± 1.60 ^c^	76.18 ± 1.46 ^b^
Total uronic acid (%)	43.88 ± 0.65 ^b^	43.32 ± 0.73 ^b^	45.7 ± 0.98 ^a^
Protein content (%)	3.50 ± 0.12 ^c^	6.08 ± 0.15^a^	4.28 ± 0.09 ^b^
M_w_ × 10^5^ (Da, error)	1.689 (±0.65%) ^c^	1.769 (±0.63%) ^b^	1.955 (±0.48%) ^a^
M_w_/M_n_ (error)	1.827 (±1.04%)	1.724 (±0.91%)	1.833 (±0.70%)
Degree of esterification (%)	43.33 ^c^	48.38 ^b^	55.02 ^a^
Constituent Monosaccharides and Molar Ratios			
Mannose	0.36	0.77	0.25
Rhamnose	0.23	0.47	0.35
Galacturonic acid	3.28	5.16	4.07
Glucose	1.00	1.00	1.00
Galactose	1.69	2.91	1.24
Xylose	0.24	0.36	0.24
Arabinose	0.93	1.86	1.01

KPS-M, kiwifruit polysaccharides (KPS) extracted by microwave-assisted extraction; KPS-U, KPS extracted by ultrasound-assisted extraction; KPS-H, KPS extracted by hot water extraction; Values represent mean ± standard deviation, and superscripts ^a^^–^^c^ differ significantly (*p* < 0.05) among KPS-M, KPS-U, and KPS-H; Statistical significances were carried out by ANOVA, followed by Duncan’s test.
